# Dietary combination of linseed and hazelnut skin as a sustainable strategy to enrich lamb with health promoting fatty acids

**DOI:** 10.1038/s41598-024-60303-3

**Published:** 2024-05-02

**Authors:** Martino Musati, Pilar Frutos, Antonino Bertino, Gonzalo Hervás, Giuseppe Luciano, Claudio Forte, Alessandro Priolo, Massimiliano Lanza, Marco Bella, Luisa Biondi, Antonio Natalello

**Affiliations:** 1 Dipartimento di Agricoltura, Alimentazione e Ambiente (Di3A), University of Catania, Via Santa Sofia 100, 95123 Catania, Italy; 2 Instituto de Ganadería de Montaña (CSIC-University of León), Finca Marzanas s/n, 24346 Grulleros, León, Spain; 3 Department of Veterinary Sciences, University of Turin, Largo Paolo Braccini 2, 10095 Grugliasco (TO), Italy

**Keywords:** By-product, Phenolic compounds, Tannins, Omega-3, Rumen, Meat, Biological techniques, Chemical biology, Biochemistry, Chemical modification

## Abstract

This study investigated the effect of the inclusion of extruded linseed and hazelnut skin on fatty acid (FA) metabolism in finishing lambs. Forty lambs were divided into 4 groups and fed for 60 d with: a conventional cereal-based diet, or the same diet with 8% of extruded linseed, or 15% of hazelnut skin, or 4% of linseed plus 7.5% of hazelnut skin as partial replacement of maize. Dietary treatments did not affect growth performances, carcass traits, and ruminal fermentation. The combined effect of linseed and hazelnut skin enriched the intramuscular fat with health promoting FA. Particularly, increases in α-linolenic acid (3.75-fold), and very long-chain n-3 poly-unsaturated FA (+ 40%) were attributed to the supplementation with linseed, rich in α-linolenic acid. In addition, increases in rumenic (+ 33%), and vaccenic (+ 59%) acids were attributed to hazelnut skin tannins modulating ruminal biohydrogenation and accumulating intermediate metabolites. The simultaneous inclusion of linseed and hazelnut skin can be a profitable strategy for enriching the intramuscular fat of lambs with health promoting FA, without adverse effects on ruminal fermentation and animal performance.

## Introduction

Improvements in the purchasing power of consumers has led to an increasing demand for healthy animal products^1^. Following public health policies, this should imply a reduction in their content of saturated fatty acids (SFA) and an increase of unsaturated ones (UFA), to lower the incidence of cardiovascular and metabolic diseases^2^.

Meat, together with milk and dairy products, are the main sources of SFA in human diet^3^. However, some animal feeding strategies (e.g., diet supplementation with oil seeds or plant oils) can be used to enhance UFA in meat at the expense of SFA, offering consumers an opportunity to modify SFA and UFA intake without relevant changes in eating habits^4^. Dietary linseed has been demonstrated to be capable of reducing SFA in meat while increasing the accumulation of potentially health-promoting n-3 polyunsaturated fatty acids (PUFA)^5,6^.

In addition to healthier animal products, consumers’ demand also requires livestock production systems to be environmentally sustainable^7^. In this respect, the inclusion of agro-industrial by-products, not edible by humans, in animal diets may be a strategy to reduce their costly disposal and the feed to food competition. Their use as feed may also improve economic sustainability by reducing feeding cost^8,9^.

Hazelnut (*Corylus avellana* L.) accounted for 11% of dried fruit global production in 2022/2023^10^. Hazelnut kernels are mainly employed in confectionary and chocolate industries^11^, where they are peeled during the roasting process, generating a considerable amount of skin to get rid of it^12^. Hazelnut skin is a fibrous by-product suitable for ruminant feeding, with a relatively high content of oleic acid (OA)^13–15^. Moreover, this skin is a source of phenolic compounds, particularly tannins, known to modulate rumen biohydrogenation (BH) and therefore reduce the accumulation of SFA and increase unsaturated FA in animal products^16,17^. A previous study with lambs fed 15% of hazelnut skin in replacement of maize found interesting results on health-promoting fatty acids (FA) in meat [i.e., a higher content of vaccenic acid (VA) and PUFA] without negative effects on growth performance^14^.

Thus, given the positive effects of both linseed and hazelnut skin in lamb diets, we hypothesized that a combination of these two ingredients could improve meat FA profile, thanks to the n-3 PUFA provided by linseed and the modulatory BH action of hazelnut skin tannins. It would also lower diet cost, presumably without negative consequences on animal performance.

The work by Mele et al.^18^, feeding lambs with diets including olive cake and linseed, might provide further support for this hypothesis, as olive cake and hazelnut skin have some similar chemical characteristics. They both contain phenolic compounds and a relatively high proportion of OA. In that assay, the reduction of the diet cost was accompanied by a higher proportion of PUFA in lamb meat without any adverse effects on animal performance.

To test our hypothesis, we conducted an experiment with lambs fed a typical concentrate-based diet for growth, in which maize and soybean meal were partially replaced with linseed and/or hazelnut skin. We studied some animal performance and rumen fermentation parameters, as well as the FA composition of rumen and abomasum digesta, and *longissimus dorsi* (LD) muscle.

## Material and methods

### Animals and diets

Experimental procedures with animals were conducted in accordance with European Union (Council Directive 2010/63/EU) legislation for the protection of animals used for experimental and other scientific purposes, being approved by the Research Ethics Committees of the University of Catania (protocol number: 82427) and conducted in accordance with ARRIVE guidelines.

Forty Valle del Belice × Comisana male lambs (2-months of age; initial body weight 17.3 kg ± SD 3.17) were selected from a local dairy sheep farm. The lambs had been weaned at the age of 45 days and fed a commercial weaning concentrate for lambs (composed of maize, wheat bran, extracted soybean meal, dehydrated alfalfa, urea, and minerals) until the start of this trial. They were taken to the experimental farm of the University of Catania, housed in individual pens (1.5 m^2^) with straw litter, and divided into four groups balanced for the initial body weight (BW). Each group received one of the following treatments (diets formulated to have comparable levels of energy and protein; see Table 1 for details):

- Control (C diet; 10 lambs): lambs received a typical maize-barley based concentrate for lamb growth.
- Linseed (L diet; 10 lambs): lambs received the control diet, with 8% of extruded linseed as partial replacement of maize and extracted soybean meal. Extruded linseed was purchased from Mazzoleni S.p.A. (Cologno al Serio, Bergamo, Italy) following the European regulation (CE n° 183/2005). During the trial, one animal from this group died due to reasons not linked to the experimental diet.
- Hazelnut (H diet; 10 lambs): lambs received the control diet, with 15% of hazelnut skin as partial replacement of maize. The level of inclusion of the by-product was chosen on the basis of previous results (see Priolo et al.^14^). Hazelnut skin was supplied by Dalma Mangimi S.p.A. (Marene, Cuneo, Italy).
- Linseed + Hazelnut (L + H diet; 10 lambs): lambs received the control diet, with 4% of extruded linseed and 7.5% hazelnut skin as partial replacement of maize.

**Table 1:** Ingredients and chemical composition of the experimental diets.

	Experimental diets^a^			
	C	L	H	L + H
Ingredients (g/kg DM)				
Maize	260	210	110	145
Barley	260	260	260	260
Alfalfa hay	200	200	200	200
Extracted soybean meal	160	130	160	160
Wheat bran	70	70	70	70
Molasses	30	30	30	30
Mineral mix	20	20	20	20
Extruded linseed	–	80	–	40
Hazelnut skin	–	–	150	75
Chemical composition (% DM)				
OM	92.2	93.2	93.1	92.9
CP	16.6	16.4	16.7	17.8
aNDF	22.5	23.5	26.4	26.1
ADF	11.3	11.8	13.5	13.5
Total phenols^b^	5.17	7.16	24.6	12.4
Total tannins^b^	1.70	3.63	15.9	6.62
Total FA	2.36	5.28	4.46	5.20
Individual fatty acid (% of total)				
16:0	19.2	12.1	11.8	13.6
18:0	3.27	3.24	2.20	4.86
* c*9 18:1 (OA)	22.0	30.6	54.3	36.5
* c*9*c*12 18:2 (LA)	51.3	32.7	28.8	27.8
* c*9*c*12*c*15 18:3 (αLNA)	2.95	19.8	1.54	15.1

*DM* dry matter, *OM* organic matter, *CP* crude protein, *aNDF* neutral detergent fibre, *ADF* acid detergent fibre, *FA* fatty acids, *OA* oleic acid, *LA* linoleic acid, *αLNA* α-linoleic acid.

^a^*C* control diet, *L* Linseed diet, *H* Hazelnut skin diet, *L* + *H* Linseed + Hazelnut skin diet.

^b^Expressed as g tannic acid equivalents/kg DM.

Experimental diets were pelleted (3 mm pellet diameter) to avoid feed selection. The animals were gradually adapted to them by progressive replacement of their commercial weaning diet over 5 day-period. Clean drinking water was always available.

Voluntary feed intakes were determined by weighing the amounts of fresh matter offered and refused by each lamb every 2–3 days. Intake was then corrected for dry matter (DM) as described below.

All animals were weighed weekly throughout the trial (Spider 3, Mettler Toledo, Columbus, OH, USA).

On day 60 on feeding trial, diets and water were removed 3 h before slaughter. Lambs were weighted and taken to a commercial abattoir (80 km far from the University farm) where they were immediately slaughtered following European regulation (Council Regulation n. 1099/2009).

### Samplings and measurements at slaughter

Individual whole ruminal and abomasal contents were collected within 10 min of slaughter and homogenized. After measuring the pH (Orion 9106; Orion Research Incorporated, Boston, MA, USA), one sample (approx. 120 mL) of each content was immediately frozen with dry ice, and then freeze-dried, and stored at – 80 °C. Another sample of rumen digesta was filtered through four cheesecloth layers and then centrifuged at 3122×*g* for 5 min (MPW-54; MPW Med. Instruments, Warsaw, Poland). Five mL of fluid was acidified with 5 mL of 0.2 *N* HCl for ammonia analysis^19^. For volatile fatty acid (VFA) determinations, another aliquot of rumen fluid (0.8 mL) was added to 0.5 mL of a deproteinizing solution (2% metaphosphoric acid containing 0.4% crotonic acid (Sigma–Aldrich, St. Louis, USA) as an internal standard, w/v in 0.5 *N* HCl). These ruminal samples for ammonia and VFA were stored at – 80 °C prior to laboratory analyses.

Following evisceration, carcasses were stored at 4 °C. After a 24-h post-mortem period, the carcasses were weighed, halved, and the LD muscle was subsequently excised from each right half. A portion of LD (approximately 100 g) was vacuum-packed and stored at – 80 °C until analysis.

### Chemical analyses

Samples of the feedstuff, which had been collected during the trial, were prepared (ISO 6498:2012) and analyzed for dry matter (DM; ISO 6496:1999), ash (ISO 5984:2022), and crude protein (ISO5983-2:2009). The neutral and acid detergent fibres (aNDF and ADF) concentrations were sequentially determined using an Ankom^2000^ fibre analyzer (Ankom Technology Methods 13 and 12, respectively; Ankom Technology Corp., Macedon, NY, USA); the former was assayed with sodium sulfite and α-amylase, and both aNDF and ADF were expressed with residual ash.

Total phenolic compounds and total tannins in feedstuffs were quantified following the Folin-Ciocalteu method developed by Makkar et al.^20^, with adaptations made by Luciano et al.^21^. Briefly, grounded feeds were extracted using acetone 70% (v/v). The concentration of total phenolic compounds was determined using Folin-Ciocalteu reagent (1 *N*) and sodium carbonate 20% (w/v). Total non-tannin phenols were treated with polyvinylpyrrolidone (PVPP) in order to precipitate tannins. Total tannins were calculated as the difference between total phenols and total non-tannin phenols. Phenolic compounds and tannins were quantified using tannic acid (Sigma–Aldrich) as a reference standard and they were expressed as mg TA equivalents/g DM.

Ammonia concentration in ruminal fluid centrifuged samples was measured spectrophotometrically (UV-1601; Shimadzu Corporation, Milan, Italy) according to Reardon et al.^19^. Ruminal VFA were determined by gas chromatography (ThermoQuest, Milan, Italy) following indications by Priolo et al.^14^.

*Longissimus dorsi* muscle was deprived of any visible fat (i.e., of the intermuscular fat), finely minced with a knife, weighted, and then homogenized with a solution of chloroform and methanol (2:1, v/v). After the evaporation of these solvents in a rotary evaporator system (Rotavapor R-114, Büchi, Flawil, Switzerland), total intramuscular fat (IMF) was determined gravimetrically.

### Fatty acid composition analyses

Feedstuff fatty acids were extracted using chloroform^22^ and converted to fatty acid methyl esters (FAME) with 2% (v/v) sulfuric acid in methanol^23^, using tridecanoic acid (13:0; Sigma-Aldrich) as an internal standard.

Rumen and abomasum digesta FA were directly converted to FAME by using a combined basic and acid methylation^24^, with nonadecanoic acid (19:0; Sigma–Aldrich) as an internal standard.

Intramuscular fat was dissolved in a mixture of hexane and 2-propanol (4:1, v/v), and 50 mg of lipids were methylated using 1 mL of sodium methoxide in methanol 0.5 *N* and 2 mL of hexane^25^. Nonadecanoic acid (19:0) was used as an internal standard.

Fatty acid methyl esters of feeds, rumen, and abomasum digesta, and muscle were separated with a gas chromatograph (ThermoQuest, Milan, Italy) equipped with a flame ionization detector (FID) and 100 m high-polar fused silica capillary column (100 m × 0.25 mm i.d.; film thickness 0.25 μm; SP-2560 fused silica, Supelco, Bellafonte, PA, USA). Total FAME were determined using a temperature gradient program at a split ratio of 1:80 and helium as carrier gas at a constant flow of 1 mL/min (for more details, see Priolo et al.^14^). The isomers *t*10 18:1 and *t*11 18:1 were further resolved in a separate analysis under isothermal condition at 165°C (adapted from Shingfield et al.^23^).

Peaks were identified based on retention time comparisons with commercially available standard mixture of FAME (Nu-Chek Prep Inc., Elysian, MN, USA; Larodan Fine Chemicals, Malmo, Sweden).

### Calculations and statistical analysis

Average daily gain was estimated as the regression slope of BW against time, using the REG procedure of the SAS software package (version 9.4; SAS Institute Inc., Cary, NC, USA).

The BH estimates (BH_FAx_) of OA (*c*9 18:1), linoleic acid (LA; *c*9*c*12 18:2), and α-linolenic acid (αLNA; *c*9*c*12*c*15 18:3) in ruminal and abomasal digesta were calculated using the equations by Oliveira et al.^26^. These estimates are based on the difference of each of these FA between diet and ruminal or abomasal digesta contents (i.e., its disappearance), and are calculated as shown below:

$${BH}_{{FA}_{x}}=\frac{({FAx}_{diet}- {FAx}_{rumen\, or\, abomasum})}{{FAx}_{diet}} \times 100$$

The BH completeness in rumen and abomasum was also estimated^27^: the higher the values of completeness in rumen and abomasum, the more complete BH resulting in 18:0 production (or, the lower the values, the higher the accumulation of BH intermediates).

$$BH \,completeness \left(\%\right)=\frac{{18:0}_{rumen\, or\, abomasum}}{\begin{array}{c}{18:0}_{diet}+\left({c9 18:1}_{diet}-{c9 18:1}_{rumen\, or \,abomasum}\right)\\ +\left({c9 c12 18:2}_{diet}-{c9 c12 18:2}_{rumen\, or\, abomasum}\right)\\ +\left({c9 c12 c15 18:3}_{diet}-{c9 c12 c15 18:3}_{rumen \,or\, abomasum}\right)\end{array}}\times 100$$

We also calculated an atherogenic index following the formula proposed by Ulbricht and Southgate^28^:

$$AI=\frac{12:0+\left(4 \times 14:0\right)+16:0}{MUFA+PUFA n-6+PUFA n-3}$$

All statistical analyses were conducted with the SAS software package. Data were analyzed with ANOVA to test the effect of the dietary treatments, using the MIXED procedure of SAS and considering the individual lambs as the experimental units. Means were separated through the pairwise differences (“pdiff”) option of the least squares means (“lsmeans”) statement of the MIXED procedure. They were adjusted for multiple comparisons using Bonferroni’s correction. Differences were declared significant at *P* < 0.05 and considered a trend toward significance at 0.05 ≤ *P* < 0.10. Least squares means are reported throughout the manuscript.

### Ethics approval

Experimental procedures with animals were conducted in accordance with European Union (Council Directive 2010/63/EU) legislation for the protection of animals used for experimental and other scientific purposes, being approved by the Research Ethics Committees of the University of Catania (protocol number: 82427).

## Results

### Animal performance

As shown in Table 2, DM intake was not affected by the treatments (*P* > 0.10). However, lambs fed the control diet ingested less total FA than the other lambs (*P* < 0.001), while aNDF and crude protein intakes were similar (*P* > 0.05). Concerning individual FA, L + H lambs consumed a greater quantity of 16:0 than the C lambs (*P* < 0.001). The daily intake of stearic acid (SA; 18:0) was higher in L + H, followed by L and finally by C and H (*P* < 0.001). Lambs fed L and L + H ingested more LA than C and H lambs (*P* = 0.001). The intake of αLNA was greater in L followed by L + H, while it was lower in C and H (*P* < 0.001). The H lambs consumed more OA compared to all the other lambs (*P* < 0.001). On the other hand, the inclusion of hazelnut skin or linseed, either individually or in combination, did not influence BW, average daily gain (ADG), feed conversion ratio (FCR), carcass weight, carcass yield, or IMF.

**Table 2:** Effect of dietary treatments on animal performances.

	Diet^a^				SED^b^	*P*-value
	C	L	H	L + H		
No. of animals	10	9	10	10		
Body weight						
Initial BW (kg)	17.9	17.2	17.0	17.2	1.51	0.938
Final BW (kg)	30.9	31.4	28.5	29.9	2.13	0.555
ADG (g/day)	227	237	193	214	24.8	0.323
Intake						
DMI (g DM/day)	831	852	862	893	77.4	0.870
aNDF intake (g/day)	186	200	227	233	19.5	0.066*
Crude protein intake (g/day)	138	140	144	159	13.3	0.354
Total FA intake (g/day)	19.6b	45.0 a	38.5 a	46.4 a	3.68	< 0.001
16:0 intake (g/day)	3.77 c	5.46 ab	4.55 bc	6.29 a	0.488	< 0.001
18:0 intake (g/day)	0.64 c	1.46 b	0.84 c	2.25 a	0.153	< 0.001
* c*9 18:1 (OA) intake (g/day)	4.32 c	13.8 b	20.9 a	17.0 b	1.42	< 0.001
* c*9*c*12 18:2 (LA) intake (g/day)	10.1 b	14.7 a	11.1 b	12.9 ab	1.11	0.001
* c*9*c*12*c*15 18:3 (αLNA) intake (g/day)	0.58 c	8.93 a	0.59 c	7.02 b	0.540	< 0.001
Feed conversion ratio (FCR)^c^	4.30	4.07	5.17	5.03	0.531	0.118
Carcass weight (kg)	14.1	14.4	13.1	14.3	1.19	0.691
Carcass yield (%)	45.4	45.9	45.6	47.7	1.82	0.572
IMF (g/100 g muscle)	2.40	2.41	1.91	2.42	0.251	0.124

a,b,c Within a row, different superscripts indicate significant differences (P < 0.05).

*BW* body weight, *ADG* average daily gain, *DMI* dry matter intake, *DM* dry matter, *aNDF* neutral detergent fibre, *FA* fatty acid, *OA* oleic acid, *LA* linoleic acid, *αLNA* α-linoleic acid, *IMF* intramuscular fat.

^a^*C* control diet, *L* Linseed diet, *H* Hazelnut skin diet, *L* + *H* Linseed + Hazelnut skin diet.

^b^*SED* standard error of the difference.

^c^Calculated as DMI/ADG.

*In the pairwise analysis, no significant differences (P > 0.10) were found after adjustment for multiple comparisons using Bonferroni’s correction.

### Rumen fermentation

Table 3 shows the effects of diet on ruminal fermentation. Ruminal pH, and ammonia and total VFA concentrations were not affected (*P* > 0.10) and, concerning molar proportion of individual VFA, only valerate showed significative effects (*P* = 0.011), being highest in L and lowest in L + H.

**Table 3:** Effect of dietary treatments on rumen and abomasum pH, and ruminal ammonia and volatile fatty acid (VFA) concentration.

	Diet^a^				SED^b^	*P*-value
	C	L	H	L + H		
pH						
Rumen	5.41	5.43	5.64	5.74	0.202	0.309
Abomasum	4.23	3.84	4.06	4.21	0.441	0.803
Ammonia (mg/L)	112	135	132	136	43.8	0.925
Total VFA (mmol/L)	128	107	110	99	15.0	0.248
VFA (mmol/100 mmol)						
Acetate	42.5	42.8	46.9	47.8	3.09	0.196
Propionate	44.2	44.0	37.8	37.2	4.07	0.167
Butyrate	7.32	7.08	10.40	10.09	1.781	0.130
* iso*-Butyrate	0.90	0.91	0.72	0.77	0.306	0.898
Valerate	3.81 ab	4.19 a	2.85 ab	2.78 b	0.482	0.011
* iso*-Valerate	1.02	0.76	0.80	1.04	0.261	0.587
Caproate	0.27	0.34	0.57	0.26	0.266	0.624
Acetate:propionate ratio	0.98	0.99	1.48	1.73	0.425	0.215

a,b Within a row, different superscripts indicate significant differences (P < 0.05).

^a^*C* control diet, *L* Linseed diet, *H* Hazelnut skin diet, *L* + *H* Linseed + Hazelnut skin diet.

^b^*SED* standard error of the difference.

### Fatty acid composition of the ruminal digesta

As shown in Table 4, the partial replacement of maize and soybean meal with linseed and hazelnut skin reduced the proportions of atherogenic FA (12:0, 14:0, and 16:0) compared to the C (*P* < 0.05). Odd (13:0, 15:0, and 17:0) and branched chain FA (15:0 *anteiso* and *iso* FA, but not 17:0 *anteiso*) (OBCFA) showed lower proportions when lambs were fed L, H, and L + H (*P* < 0.001). In line with this, the summation of OBCFA was significantly higher in C lambs compared to all the others (*P* < 0.001).

**Table 4:** Effect of dietary treatments on rumen digesta fatty acids (g/100 g total fatty acids).

	Diet^a^				SED^b^	*P*-value
	C	L	H	L + H		
12:0	0.54 a	0.37 b	0.30 bc	0.23 c	0.041	< 0.001
13:0	0.19 a	0.09 b	0.09 b	0.09 b	0.015	< 0.001
14:0	2.07 a	1.69 ab	1.09 b	0.93 b	0.325	0.003
*c*9 14:1	0.88 a	0.43 b	0.48 b	0.44 b	0.062	< 0.001
*t*9 14:1	0.11 x	0.05 y	0.06 y	0.07 y	0.021	0.045
15:0	1.31 a	0.65 b	0.76 b	0.70 b	0.079	< 0.001
15:0* anteiso*	1.59 a	0.85 b	0.73 b	0.95 b	0.137	< 0.001
15:0 *iso*	0.60 a	0.25 b	0.32 b	0.29 b	0.081	< 0.001
*t*10 15:1	0.22	0.12	0.19	0.15	0.041	0.082*
16:0	16.3 x	12.9 y	13.2 y	13.5 xy	1.25	0.031
*c*9 16:1	0.27	0.26	0.11	0.15	0.091	0.225
*t*9 16:1	0.15	0.09	0.05	0.07	0.043	0.127
17:0	0.60 a	0.37 ab	0.27 b	0.27 b	0.085	< 0.001
17:0* anteiso*	0.07	0.06	0.06	0.07	0.021	0.844
*c*9 17:1	0.20 a	0.14 ab	0.08 b	0.11 ab	0.041	0.035
18:0 (SA)^c^	32.0	34.7	38.1	38.3	3.79	0.283
*c*6 18:1	1.62 b	3.69 a	1.41 b	3.21 a	0.462	< 0.001
*c*9 18:1 (OA)^c^	8.91 b	10.9 b	19.6 a	13.7 b	1.75	< 0.001
*c*11 18:1	1.89 a	1.30 b	1.30 b	1.24 b	0.140	< 0.001
*t*6 + 7 + 8 18:1	0.97	1.50	1.39	1.49	0.284	0.204
*t*9 18:1	0.48	0.88	0.66	0.72	0.204	0.277
*t*10 18:1	11.0 x	9.39 xy	5.27 y	5.59 y	2.15	0.023
*t*11 18:1 (VA)^c^	2.03	2.05	1.69	2.44	0.554	0.596
*c*9*c*12 18:2 (LA)^c^	11.8 a	7.71 b	7.93 b	7.56 b	1.19	0.002
*c*9*t*11 18:2 (RA)^c^	0.26 a	0.28 a	0.16 b	0.23 ab	0.032	0.005
*t*10*c*12 18:2	< 0.01	0.04	< 0.01	0.01	0.027	0.408
*t*9*t*12 18:2	0.46	0.48	0.21	0.33	0.125	0.116
*c*6*c*9*c*12 18:3	0.45 a	0.34 b	0.29 b	0.34 b	0.037	< 0.001
*c*9*c*12*c*15 18:3 (αLNA)^c^	0.74 c	4.81 a	0.45 c	2.95 b	0.398	< 0.001
*t*7 19:1	0.20 b	0.35 a	0.05 c	0.19 b	0.037	< 0.001
*t*10 19:1	0.47 c	2.15 a	0.16 c	1.35 b	0.252	< 0.001
*c*11 20:1	0.21 a	0.18 ab	0.12 b	0.15 ab	0.032	0.047
*c*11*c*14 20:2	0.11 a	0.05 b	0.05 b	0.05 b	0.008	< 0.001
20:3 n-3	0.06 x	0.02 y	0.03 xy	0.04 xy	0.014	0.049
22:0	0.30 a	0.20 b	0.16 b	0.20 b	0.024	< 0.001
23:0	0.09 x	0.05 xy	0.04 xy	0.03 y	0.023	0.067
24:0	0.28 a	0.18 b	0.17 b	0.20 b	0.023	< 0.001
*c*9 24:1	0.01	< 0.01	< 0.01	0.01	0.005	0.318
22:5 n-3	0.13 a	0.07 b	0.09 b	0.10 ab	0.014	0.002
SFA^c^	56.0	52.3	55.3	55.8	3.86	0.770
MUFA^c^	29.7	33.5	32.7	31.1	2.86	0.537
PUFA^c^	14.0 a	13.8 a	9.20 b	11.6 ab	1.48	0.007
OBCFA^c^	4.46 a	2.36 b	2.28 b	2.42 b	0.201	< 0.001
Ratio *t*10/*t*11 18:1	7.77	6.10	5.13	3.47	2.45	0.348

a,b,c Within a row, different superscripts indicate significant differences (P < 0.05).

x,y Within a row, different superscripts indicate a trend towards significant differences (0.05 ≤ P < 0.10), either for the general statistical model or after Bonferroni’s correction.

^a^*C* control diet, *L* Linseed diet, *H* Hazelnut skin diet, *L + H* Linseed + Hazelnut skin diet.

^b^*SED* standard error of the difference.

^c^*SA* stearic acid, *OA* oleic acid, *VA* vaccenic acid, *LA* linoleic acid, *RA* rumenic acid, *αLNA* α-linoleic acid, *SFA* saturated fatty acid, *MUFA* monounsaturated fatty acid, *PUFA* polyunsaturated fatty acid, *OBCFA* odd- and branched-chain fatty acid.

*In the pairwise analysis, no significant differences (P > 0.10) were found after adjustment for multiple comparisons using Bonferroni’s correction.

Diet did not affect SA and VA proportions in the rumen liquor (*P* > 0.10). The concentration of OA was higher in H than in the other treatments (*P* < 0.001), with a twofold increase compared to C. The proportion of *t*10 18:1 showed significant effect (*P* = 0.023), but Bonferroni’s adjustment reduced it to a tendency to differences between C and treatments with hazelnut skin (i.e., H and L + H). The LA content was significantly greater in rumen fluid from control animals compared to all other treatments (*P* = 0.002), while the rumen digests from H-fed lambs showed lower rumenic acid (RA; *c*9*t*11 18:2) concentration than C and L (*P* = 0.005). The proportion of αLNA in L was 10.7-fold higher than in H, 6.5-fold than in C, and 1.6-fold than in L + H (*P* < 0.001). Concerning FA groups (summations), no effect of diet was found on SFA and monounsaturated FA (MUFA; *P* > 0.10), but feeding H lowered PUFA proportion compared to C and L (*P* = 0.007). The ratio of *t*10/*t*11 18:1 was not affected by treatments (*P* > 0.10).

### Fatty acid composition of the abomasal digesta

The FA composition of the abomasal digesta is shown in Table 5. In general, the effects of dietary treatments were similar to those observed in the rumen. In fact, proportions of FA with 17 or fewer carbons, OA, *t*10 18:1, VA, LA, αLNA, SFA, MUFA, OBCFA, and *t*10/*t*11 ratio followed the same pattern between diets as in the rumen. However, there were a few variations, lambs fed L + H tended to have a higher SA proportion than C lambs (*P* = 0.05), and RA was not significantly affected by dietary inclusion of linseed and/or hazelnut skin (*P* > 0.10). The summation of total PUFA showed significant variation (*P* = 0.032), but Bonferroni’s adjustment reduced it to a tendency (*P* < 0.10) to differences only between C and H.

**Table 5:** Effect of dietary treatments on abomasum digesta fatty acids (g/100 g total fatty acids).

	Diet^a^				SED^b^	*P*-value
	C	L	H	L + H		
12:0	0.62 a	0.40 b	0.25 bc	0.21 c	0.056	< 0.001
13:0	0.21 a	0.10 b	0.07 b	0.07 b	0.020	< 0.001
14:0	2.58 a	1.25 b	1.10 b	0.87 b	0.222	< 0.001
*c*9 14:1	0.93 a	0.39 b	0.42 b	0.41 b	0.059	< 0.001
*t*9 14:1	0.12 a	0.05 b	0.06 b	0.04 b	0.019	< 0.001
15:0	1.07 a	0.53 b	0.42 b	0.50 b	0.095	< 0.001
15:0* anteiso*	1.66 a	0.84 b	0.63 b	0.82 b	0.149	< 0.001
15:0 *iso*	0.54 a	0.18 b	0.23 b	0.19 b	0.071	< 0.001
*t*10 15:1	0.20 a	0.10 b	0.11 b	0.09 b	0.025	< 0.001
16:0	17.5 a	11.8 b	12.3 b	11.8 b	0.65	< 0.001
*c*9 16:1	0.21 x	0.12 xy	0.14 xy	0.11 y	0.041	0.053
*t*9 16:1	0.16 a	0.03 b	0.06 b	0.07 b	0.037	0.005
17:0	0.71 a	0.29 b	0.34 b	0.33 b	0.083	< 0.001
17:0* anteiso*	0.04	0.03	0.07	0.04	0.018	0.139
*c*9 17:1	0.12 x	0.04 y	0.07 xy	0.09 xy	0.028	0.059
18:0 (SA)^c^	32.7 y	39.8 xy	40.0 xy	43.5 x	4.18	0.050
*c*6 18:1	1.65 b	4.57 a	1.34 b	3.55 a	0.541	< 0.001
*c*9 18:1 (OA)^c^	8.93 b	8.66 b	20.1 a	12.1 b	1.47	< 0.001
*c*11 18:1	2.00 a	1.38 b	1.32 b	1.20 b	0.167	< 0.001
*t*6 + 7 + 8 18:1	1.04 b	1.78 a	1.62 b	1.86 a	0.276	0.010
*t*9 18:1	0.52 b	0.78 ab	0.79 ab	0.88 a	0.134	0.032
*t*10 18:1	10.6 x	8.86 xy	5.11 y	6.30 xy	2.152	0.042
*t*11 18:1 (VA)^c^	1.88	2.24	1.42	2.08	0.479	0.359
*c*9*c*12 18:2 (LA)^c^	10.2ª	6.69 b	7.17 ab	5.92 b	1.21	0.002
*c*9*t*11 18:2 (RA)^c^	0.21	0.20	0.18	0.22	0.039	0.659
*t*10*c*12 18:2	< 0.01	< 0.01	0.01	< 0.01	0.007	0.650
*t*9*t*12 18:2	1.02	0.67	0.54	0.46	0.282	0.155
*c*6*c*9*c*12 18:3	0.40	0.36	0.29	0.38	0.052	0.180
*c9c12c*15 18:3 (αLNA)^c^	0.54 c	4.39 a	0.37 c	2.40 b	0.443	< 0.001
*t*7 19:1	0.07 b	0.13 ab	0.03 b	0.20 a	0.046	0.004
*t*10 19:1	0.25 c	2.37 a	0.18 c	1.22 b	0.350	< 0.001
*c*11 20:1	0.17	0.15	0.12	0.13	0.033	0.365
*c*11*c*14 20:2	0.09 a	0.05 b	0.07 b	0.05 b	0.007	< 0.001
22:0	0.26 a	0.20 b	0.17 b	0.22 ab	0.022	0.001
20:3 n-3	0.04	0.02	0.02	0.03	0.018	0.571
23:0	0.04	0.04	0.02	0.04	0.019	0.703
24:0	0.22	0.20	0.19	0.19	0.039	0.883
*c*9 24:1	0.01	< 0.01	0.01	0.01	0.006	0.196
22:5 n-3	0.09	0.07	0.09	0.11	0.021	0.289
SFA^c^	58.2	55.7	55.9	58.8	4.19	0.805
MUFA^c^	28.9	31.7	33.0	30.3	2.95	0.518
PUFA^c^	12.6 x	12.4 xy	8.73 y	9.60 xy	1.61	0.032
OBCFA^c^	4.27 a	2.02 b	1.78 b	2.00 b	0.263	< 0.001
Ratio *t*10/*t*11 18:1	7.05	4.59	5.01	4.51	2.32	0.593

a,b,c Within a row, different superscripts indicate significant differences (P < 0.05).

x,y Within a row, different superscripts indicate a trend towards significant differences (0.05 ≤ P < 0.10), either for the general statistical model or after Bonferroni’s correction.

^a^*C* control diet, *L* Linseed diet, *H* Hazelnut skin diet, *L + H* Linseed + Hazelnut skin diet.

^b^*SED* standard error of the difference.

^c^*SA* stearic acid, *OA* oleic acid, *VA* vaccenic acid, *LA* linoleic acid, *RA* rumenic acid, *αLNA* α-linoleic acid, *SFA* saturated fatty acid, *MUFA* monounsaturated fatty acid, *PUFA* polyunsaturated fatty acid, *OBCFA* odd- and branched-chain fatty acid.

### Biohydrogenation indices

Biohydrogenation indices for OA, LA, and αLNA, estimated with rumen and abomasum data, are given in Table 6. Only the BH index of αLNA in the rumen, which had higher values in L + H compared to H, and of the OA in the abomasum, which was higher in L compared to C, showed statistically significant differences (*P* = 0.026 and 0.009, respectively). Regarding the index of BH completeness, there was a significant effect of dietary treatments in the calculations with rumen data (*P* = 0.005), with a higher value in H compared to C and L. When the index of BH completeness was calculated with abomasum data, the value in H was higher than that in the control (*P* = 0.010).

**Table 6:** Effect of dietary treatments on the biohydrogenation (%) and biohydrogenation completeness (%) indices in rumen and abomasal digesta.

	Diet^a^				SED^b^	*P*-value
	C	L	H	L + H		
Biohydrogenation^c^ (%)						
*c*9 18:1 (OA)^d^						
Rumen	55.2	60.0	59.4	58.7	6.22	0.861
Abomasum	54.6 b	69.6 a	59.3 ab	64.7 ab	4.58	0.009
*c*9*c*12 18:2 (LA)^d^						
Rumen	74.6	73.7	69.1	70.1	3.33	0.274
Abomasum	77.7	77.7	72.7	77.2	3.41	0.387
*c*9*c*12*c*15 18:3 (αLNA)^d^						
Rumen	72.2 ab	73.0 ab	67.6 b	78.6 a	3.52	0.026
Abomasum	79.5	75.9	74.1	83.0	4.37	0.159
Completeness^e^ (%)						
Rumen	62.3 b	64.0 b	76.8 a	72.9 ab	4.47	0.005
Abomasum	62.8 b	66.2 ab	77.5 a	73.5 ab	4.77	0.010

a,b Within a row, different superscripts indicate significant differences (P < 0.05).

^a^*C* control diet, *L* Linseed diet, *H* Hazelnut skin diet, *L + H* Linseed + Hazelnut skin diet.

^b^*SED* standard error of the difference.

^c^Calculated as reported by Oliveira et al.^26^.

^d^*OA* oleic acid, *LA* linoleic acid, *αLNA* α-linoleic acid.

^e^Calculated as reported by Alves et al.^27^.

### Intramuscular fatty acid composition

Table 7 reports the effects of the diet on intramuscular FA composition. Feeding lambs with H reduced the proportions of 15:0 compared to C and L (*P* = 0.009), while 16:0 and *c*9 16:1 concentrations were lower in H than in C (*P* = 0.010 and 0.018, respectively). Consumption of hazelnut skin (H and L + H) resulted in a lower concentration of 17:0 compared to C (*P* = 0.001), while its inclusion, as well as that of linseed reduced the proportion of 17:0 *anteiso* (*P* < 0.001) but not of 17:0 *iso* (*P* > 0.10). Consequently, the OBCFA was higher in IMF of C lambs compared to H and L + H (*P* < 0.001). Although SA concentration was significantly affected by diet (*P* = 0.042), Bonferroni’s adjustment for multiple comparisons did not discriminate experimental groups. Oleic acid concentration was not affected by diet (*P* > 0.10), and the accumulation of VA and RA was higher in the muscle of lambs in the L + H treatment than in the control (*P* < 0.05). Animals in the L treatment had more *t*10 18:1 content than H and L + H, but not than C (*P* = 0.001). Lambs fed hazelnut skin had higher concentration of LA in muscle than L and L + H (*P* = 0.006). When compared to C and H, the αLNA proportion was 4.9-fold higher in L and 3.75-fold higher in L + H (*P* < 0.001). As for FA groups, the replacement of maize with hazelnut skin (H) tended to increase the proportion of PUFA compared to all other treatments, including the combination L + H (*P* = 0.080). Nevertheless, SFA and MUFA were not modified by diet inclusion of H and/or L (*P* > 0.10). Feeding linseed (L and L + H) lowered the proportion of PUFA n-6 in muscle compared to H (*P* = 0.004), while increased PUFA n-3 compared to L + H, and followed by C and H with the lowest values (*P* < 0.001). Consequently, the PUFA n-6/n-3 ratio was significantly higher in C and H lambs compared to L + H and L (*P* < 0.001). Although no dietary treatment was significantly different from the control, the atherogenic index tended to be higher in L + H compared to H (P = 0.061). The dietary inclusion of H lowered the desaturase index [calculated as *c*9 17:1 / (17:0 + *c*9 17:1)], but the other treatments (i.e., L and L + H) did not differ from the control (*P* = 0.021).

**Table 7:** Effect of dietary treatments on muscle fatty acids (g/100 g total fatty acids).

	Diet^a^				SED^b^	*P*-value
	C	L	H	L + H		
10:0	0.15	0.17	0.16	0.18	0.018	0.172
12:0	0.17	0.17	0.16	0.19	0.017	0.480
14:0	2.62	2.63	2.35	2.79	0.209	0.195
*c*9 14:1	0.12	0.13	0.11	0.14	0.017	0.353
*t*9 14:1	0.01	0.01	< 0.01	0.01	0.002	0.162
15:0	0.43 a	0.43 a	0.32 b	0.36 ab	0.037	0.009
15:0 *anteiso*	0.09	0.09	0.07	0.09	0.010	0.094*
15:0 *iso*	0.05	0.05	0.04	0.05	0.010	0.523
*t*10 15:1	0.10	0.09	0.10	0.10	0.010	0.548
16:0	22.9 a	22.6 ab	21.0 b	22.2 ab	0.55	0.010
*c*9 16:1	1.73 a	1.65 ab	1.31 b	1.55 ab	0.134	0.018
*t*9 16:1	0.01 x	0.02 xy	0.02 xy	0.03 y	0.006	0.041
17:0	1.68 a	1.45 ab	1.11 b	1.12 b	0.157	0.001
17:0 *anteiso*	0.53 a	0.45 b	0.37 c	0.42 bc	0.024	< 0.001
17:0 *iso*	0.33	0.29	0.30	0.29	0.030	0.432
*c*9 17:1	1.18 a	0.93 ab	0.61 c	0.67 bc	0.111	< 0.001
18:0 (SA)^c^	12.0	12.0	13.5	13.5	0.70	0.042*
*c*6 18:1	0.30 c	0.62 ab	0.47 bc	0.75 a	0.070	< 0.001
*c*9 18:1 (OA)^c^	38.6	35.7	38.3	37.4	1.32	0.150
*c*11 18:1	1.89 a	1.81 ab	1.85 a	1.49 b	0.123	0.007
*t*6 + 7 + 8 18:1	0.13 b	0.26 a	0.24 a	0.34 a	0.041	< 0.001
*t*9 18:1	0.29 b	0.38 ab	0.41 a	0.47 a	0.043	0.001
*t*10 18:1	3.06 ab	4.71 a	2.02 b	2.18 b	0.673	0.001
*t*11 18:1 (VA)^c^	0.35 b	0.64 ab	0.57 ab	0.85 a	0.118	0.001
*c*9*c*12 18:2 (LA)^c^	7.23 ab	6.48 b	9.47 a	7.18 b	0.837	0.006
*c*9*t*11 18:2 (RA)^c^	0.32 b	0.42 ab	0.37 ab	0.48a	0.055	0.030
*c*6*c*9*c*12 18:3	0.10 a	0.07 bc	0.10 ab	0.07 c	0.009	0.002
*c*9*c*12*c*15 18:3 (αLNA)^c^	0.40 c	1.96 a	0.41 c	1.50 b	0.111	< 0.001
*t*7 19:1	0.13 b	0.56 a	0.12 b	0.45 a	0.054	< 0.001
20:0	0.08 b	0.08 ab	0.09 ab	0.10 a	0.009	0.043
*c*11 20:1	0.14	0.14	0.17	0.15	0.012	0.104
*c*11*c*14 20:2	0.09 a	0.05 b	0.10 a	0.07 ab	0.013	0.003
20:3 n-6	0.19	0.16	0.21	0.15	0.036	0.074*
20:5 n-3	0.13 b	0.40 a	0.17 b	0.30 a	0.044	< 0.001
22:4 n-6	0.18 a	0.10 b	0.20 a	0.09 b	0.020	< 0.001
22:5 n-6	0.05 b	0.04 b	0.09 a	0.05 b	0.015	0.003
22:5 n-3	0.33 b	0.54 a	0.40 ab	0.46 a	0.053	0.002
22:6 n-3	0.10 b	0.15 ab	0.13 ab	0.17 a	0.022	0.028
23:0	0.15	0.13	0.18	0.14	0.023	0.227
SFA^c^	41.1	40.6	39.6	41.4	0.82	0.158
MUFA^c^	48.0	47.7	46.3	46.6	1.30	0.488
PUFA^c^	10.9 y	11.8 xy	14.0 x	12.0 xy	1.24	0.080
OBCFA^c^	3.26 a	2.90 ab	2.38 b	2.47 b	0.188	< 0.001
∑ n-3	0.95 c	3.06 a	1.11 c	2.42 b	0.207	< 0.001
∑ n-6	7.84 ab	6.89 b	10.17 a	7.61 b	0.891	0.004
Ratio n-6/n-3	8.30 a	2.24 b	9.21 a	3.14 b	0.454	< 0.001
Ratio PUFA/SFA	0.27 y	0.29 xy	0.36 x	0.29 xy	0.033	0.051
AI^d^	0.57 xy	0.56 xy	0.51 y	0.58 x	0.028	0.061
Ratio *c*9 17:1/(17:0 + *c*9 17:1)	0.41 a	0.39 ab	0.35 b	0.38 ab	0.046	0.021

a,b,c Within a row, different superscripts indicate significant differences (P < 0.05).

x,y Within a row, different superscripts indicate a trend towards significant differences (0.05 ≤ P < 0.10), either for the general statistical model or after Bonferroni’s correction.

^a^*C* control diet, *L* Linseed diet, *H* Hazelnut skin diet, *L + H* Linseed + Hazelnut skin diet.

^b^*SED* standard error of the difference.

^c^*SA* stearic acid, *OA* oleic acid, *VA* vaccenic acid, *LA* linoleic acid, *RA* rumenic acid, *αLNA* α-linoleic acid, *SFA* saturated fatty acid, *MUFA* monounsaturated fatty acid, *PUFA* poly-unsaturated fatty acid, *OBCFA* odd- and branched-chain fatty acid, *AI* atherogenic index.

^d^Calculated as reported by Ulbricht and Southgate^28^.

*In the pairwise analysis, no significant differences (P > 0.10) were found after adjustment for multiple comparisons using Bonferroni’s correction.

## Discussion

Maize and soybean are both widely used for food, feed, and fuel production^29^. Their substitution in animal diets by agro-industrial by-products may reduce not only the food-feed-fuel competition but also the feeding cost of ruminant production. In this study, we partially replaced dietary maize and soybean meal, which are common dietary ingredients for growing lambs in intensive systems^30,31^, with the by-product hazelnut skin and with linseed. Our aim was to test the hypothesis that a combination of these two ingredients may improve meat FA composition without having a negative impact on lambs’ performance. In addition, the inclusion of the by-product would, if not reduce the cost of the diet, at least maintain it despite the high price of the linseed.

The experiment included, besides the control, one treatment with the L + H combination and two individual treatments with either L or H. However, since there are numerous papers in the literature on the effect of supplementing the diet of lambs with αLNA (e.g., Andrés et al.^5^; Bessa et al.^6^; Nguyen et al.^32^), results related to linseed feeding will be discussed only briefly. On the other hand, there are very few scientific studies on the use of hazelnut skin as feed for ruminants^15,33^ and, to the best of our knowledge, only one on its use as feed for growing lambs^14^. Therefore, results about the effects of the dietary inclusion of hazelnut skin will be discussed in more detail. In any case, this section will focus particularly on the L + H treatment, in order to either accept or reject our hypothesis.

### Animal performance and ruminal fermentation

Previous studies have shown that the inclusion of up to 10% extruded linseed in the diet of lambs did not affect intake and growth performance^4^ and carcass characteristics^34^. Priolo et al.^14^ reported that growth and intake were similar in lambs fed 15% hazelnut skin or a conventional diet, but the former showed a higher FCR. Our results for the combination of L + H were consistent with these findings, although differences in FCR did not reach statistical significance (*P* = 0.118). Only animals fed the H diet showed a tendency (*P* = 0.058) to have a higher FCR than the control, which could be in line with lower values, although only numerically, of ADG and IMF in this H treatment. However, the combination L + H compensated for the numerical worsening in terms of ADG, FCR, and IMF caused by the hazelnut skin. Different batches of hazelnut skin may explain small divergences in animal performance. Indeed, a common problem of all agro-industrial by-products is the variability of their chemical composition, including the content of tannins^9,13^. High contents of these compounds can be a limiting factor in their use. For example, according to Shakeri^35^, the inclusion of 30% of pistachio by-products in the diet of lambs (corresponding to 22.8 g tannic acid equivalents/kg DM) had a negative effect on growth performance. However, when four different tannin extracts were supplemented at a concentration of 40 g/kg DM to lambs’ diet, adverse impact on animal performance was observed only with the chestnut extract and not with the other treatments^36^. Therefore, it is difficult to establish the threshold at which tannins may cease to be beneficial and become harmful. This threshold will depend not only on interactions between doses, tannin types and basal diets, but also on the use of different standards and methods of tannin analysis^37,38^.

Regarding effects on ruminal fermentation, the literature is still unclear. For instance, in dairy cows, while Martin et al.^39^ reported that increasing linseed levels in a maize silage-based diet enhanced propionate and reduced butyrate and acetate, in contrast to total VFA production and individual molar concentrations observed by Doreau et al.^40^. In lambs, 15% hazelnut skin reduced the proportion of butyrate and valerate, without affecting other rumen fermentation parameters^14^. This is very similar to present results, but no significant variation was observed in butyrate (*P* = 0.130). Under in vitro conditions, Niderkorn et al.^41^ also reported that a substrate containing 8.2% hazelnut skin and sainfoin reduced the concentration of valerate. In the present experiment, only valerate concentration was lower in L + H compared to L. This reduction in valerate would be consistent with the studies cited above. Although it was not supported by a reduction in ammonia, it would reflect the known inhibition of ruminal protein degradation by tannins^42^.

### Fatty acid metabolism

Ruminant-derived foods are rich in SFA and poor in n-3 PUFA^43^, due to rumen microbial BH of dietary UFA^44^. On this basis, linseed has been extensively studied to increase the concentration of health promoting FA, such as very long-chain n-3 PUFA, RA, and VA^32,45^, in meat^18,34^. Dietary tannins have also been used to this aim, as they can impair the extent of BH and favor the accumulation of UFA^16,46^. Thanks to its polyphenol content, hazelnut skin has been successfully incorporated into ruminant diets to modulate the rumen microbiota^47^ and the FA profile of foods^14,15,33^.

Partial replacement of maize with L + H resulted in an improvement of the meat UFA composition, with increases in αLNA, very long-chain n-3 PUFA, VA, and RA. Supplementation with linseed, rich in αLNA, led us to expect higher contents of both αLNA^18^ and very long-chain n-3 PUFA^32,34^ in meat. However, the latter is not always observed (e.g., Mele et al.^18^, Urrutia et al.^4^). In the present study, proportions of eicosapentaenoic acid (EPA, 20:5 n-3), docosapentaenoic acid (DPA, 22:5 n-3), and docosahexaenoic acid (DHA, 22:6 n-3) in muscle were significantly higher in lambs fed L or L plus H diets. This points to the effect of linseed but also to a potential influence of hazelnut skin tannins on the activity of elongase enzymes, responsible for the conversion of αLNA in EPA, DPA, and DHA in animal tissues^48^. The presence of very long-chain n-3 PUFA in animal products may have health benefits for consumers, including anti-inflammatory or cardiovascular properties^49^. Furthermore, the high proportion of total n-3 PUFA lowered the n-6/n-3 ratio below 4, which is the value recommended for the prevention of cardiovascular diseases^50^. Yet, some studies suggest that strategies to improve meat fat healthiness should increase total PUFA, including n-3 and n-6^51^. Both n-3 and n-6 are considered in the atherogenic index, which was not different from that of the control animals.

Other UFA with beneficial effects on human health, such as anticarcinogenic and antitumoral^2,52^, are RA and VA. The highest proportion of these FA was detected in L + H lambs. Rumenic acid comes from two sources. The first, and minor, one is BH of dietary LA^53^. However, the supply of LA in the L + H diet was lower than in the C, and the BH indices of LA in the rumen and abomasum were similar between treatments. The second origin is in the muscle, due to desaturation of VA, another intermediate of rumen BH, by stearoyl-CoA desaturase^23^. Nevertheless, rumen and abomasum VA concentrations did not differ among treatments and the desaturation index [calculated as *c*9 17:1/(17:0 + *c*9 17:1) ratio according to Bessa et al.^43^] was also similar, except in H lambs. Concentrations of VA were significantly higher in the intramuscular fat of L + H lambs. This is in line with findings reported by Priolo et al.^14^ when feeding lambs with 15% hazelnut skin, and by Berthelot et al.^54^ when linseed was used. We do not have a solid explanation for certain apparent inconsistencies between some of these results. However, it is important to note that direct comparisons of the results observed in the rumen, abomasum and muscle cannot be made. In fact, ruminal digestion processes are dynamic, but the sampling for FA profile can only be performed at a specific point in time, which obliges to cautious interpretation. The abomasal FA profile derives from a continuous flow of what actually comes out of the rumen and can be transferred to the intramuscular fat^55^. Finally, meat FA profile is the result of the whole feeding and metabolic processes. Although a greater consistency between abomasum and muscle FA profiles may be expected, variations due to lipid metabolism in muscle (e.g., desaturation reactions) cannot be ignored.

Moreover, it is probably worth mentioning that the higher concentration of RA, VA, and n-3 PUFA in L + H lambs would made the intramuscular fat comparable to that of grass-fed animals^56^, with a greater accumulation of these FA that are typical of lambs fed on pasture.

Regarding other *trans* 18:1 FA, *t*10 18:1 was the main *trans* FA in all treatments, consistent with the very low forage:concentrate ratio of diets used in intensive systems^57^. On the other hand, grazing or forage rich diets result in higher contents of *t*11 18:1 (VA) in animal products^56^, more comparable to values in lambs fed L + H. Low forage diets are known to alter rumen microbiota and lead to a shift of the BH pathways, with the formation of *t*10 at the expense of *t*11 18:1, which is called the “*trans*-10 shift”^27^. The accumulation of a large amount of *t*10 18:1 in animal products is associated to a higher risk of coronary heart diseases^58^ and therefore undesirable. In the present study, the increase in intramuscular *t*10 18:1 in response to linseed supplementation (in line with Bessa et al.^6^) was decreased by the incorporation of tannin-containing hazelnut skin (in line with Carreño et al.^59^ and Frutos et al.^16^), although the *t*10/*t*11 18:1 ratio was not statistically affected in ruminal and abomasal digesta. Regarding other *trans* 18:1, diets containing linseed and/or hazelnut skin (L, H, and L + H) showed higher proportions of *t*6 + 7 + 8, and *t*9 18:1 in meat, even though this was not observed in rumen and abomasum. These *trans-*18:1 can derive from numerous BH pathways, including OA isomerization^60^. In our experiment, L, H, and L + H diets provided more OA than the control, but its intramuscular accumulation did not differ among treatments. This may be accounted for by two counteracting processes: biohydrogenation of OA, as indicated by the BH index, and desaturase activity converting SA in OA in the muscle.

With regard to PUFA, despite the high amount supplied by linseed, their content in meat of L + H was not as high as expected. Nevertheless, according to the scientific literature, dietary linseed might enhance (e.g., Urrutia et al.^4^) or not (e.g., Andrés et al.^5^, Berthelot et al.^54^, Realini et al.^34^) total PUFA proportion in meat. Comparisons between studies are difficult due to interactions of types of lipid source and basal diets with ruminal BH processes^6^. In our work, only lambs fed the H diet tended to reduce BH and have higher meat PUFA proportion, which agrees with the results previously observed by Priolo et al.^14^ under similar conditions. However, although there was no significant difference in intramuscular fat (*P* = 0.124), the numerical value in H (1.91%) was lower than in the other 3 treatments, where it was very stable (2.40, 2.41, and 2.42% for C, L, and L + H). When we quantified FA in muscle (Suppl. Table S1), we confirmed that L + H had more PUFA than C and H (192 vs. 142 and 146 mg/100 g of muscle, respectively). Furthermore, considering that L and C lambs had no different content of PUFA, it may be hypothesized that tannins supplied by H probably protected PUFA supplied by L from ruminal BH, resulting in a greater accumulation of PUFA in the muscle of L + H. Quantification of FA in muscle (Suppl. Table S1) also confirmed the most relevant results observed as proportions, such as higher contents of total n-3 PUFA, VA, and RA (Table 7).

Finally, odd- and branched-chain FA, which originate from cellular membranes of ruminal bacteria^61^, have been demonstrated to be sensitive to the presence of dietary UFA^62^ and tannins^27^. This would explain the lower concentrations that we detected in lambs fed diets containing linseed and/or tannin-containing hazelnut skin, not only in rumen and abomasum, but also in muscle. This effect attributable to tannins had been found in vitro and in vivo when hazelnut skin is included in the diet^14,33^.

## Conclusion

The inclusion of 4% of extruded linseed and 7.5% of hazelnut skin as a partial replacement of maize (L + H treatment) can be a profitable strategy for improving the fatty acid profile of the meat of fattening lambs, without adverse effects on ruminal fermentation and animal performance. The combined effects of linseed and hazelnut skin allow the enrichment of intramuscular fat with health promoting FA, such as α-linolenic acid, very long-chain n-3 PUFA, rumenic acid, and vaccenic acid. Our results suggest that tannins in hazelnut skin modulate the BH process of dietary PUFA provided by linseed, protecting them from complete biohydrogenation and favoring accumulation of desirable intermediates. Moreover, hazelnut skin tannins hinder the occurrence of the *trans*-10 shift in the rumen. The use of hazelnut skin as feed may be a good strategy to reduce not only the feed-food-fuel competition but also the cost of ruminant diets. Further research would be interesting to assess other quality traits of the meat of lambs fed with n-3 PUFA sources and hazelnut skin, such as oxidative stability during shelf-life and organoleptic characteristics.

### Supplementary Information

Supplementary Table 1.

## Data Availability

The datasets used and/or analysed during the current study available from the corresponding author on reasonable request.

## Abbreviations

αLNA: α-Linolenic acid
aNDF: Amylase neutral detergent fibre
ADF: Acid detergent fibre
ADG: Average daily gain
BH: Biohydrogenation
BW: Body weight
C: Control treatment
DM: Dry matter
DHA: Docosahexaenoic acid
DPA: Docosapentaenoic acid
EPA: Eicosapentaenoic acid
FA: Fatty acid
FAME: Fatty acid methyl ester
FCR: Feed conversion ratio
FID: Flame ionization detector
H: Hazelnut skin treatment
IMF: Intramuscular fat
L: Linseed treatment
LA: Linoleic acid
LD: *longissimus dorsi*
L + H: Linseed + hazelnut skin treatment
MUFA: Monounsaturated fatty acid
OA: Oleic acid
OBCFA: Odd- and branched-chain fatty acid
PUFA: Polyunsaturated fatty acid
RA: Rumenic acid
SA: Stearic acid
SFA: Saturated fatty acid
UFA: Unsaturated fatty acid
VA: Vaccenic acid
VFA: Volatile fatty acid
