# Supplementary material for: Dietary combination of linseed and hazelnut skin as a sustainable strategy to enrich lamb with health promoting fatty acids
Source: Sci Rep. 2024 May 2;14:10133. doi: 10.1038/s41598-024-60303-3 (PMC11066009; doi:10.1038/s41598-024-60303-3)
Supplement: Supplementary file 1 — Supplementary Table 1. [file 41598_2024_60303_MOESM1_ESM.docx]

**SUPPLEMENTAL MATERIAL**

**Dietary combination of linseed and hazelnut skin as a sustainable strategy to enrich lamb meat with health promoting fatty acids**

Martino Musati^1^, Pilar Frutos^2^, Antonino Bertino^1^, Gonzalo Hervás^2^*, Giuseppe Luciano^1^, Claudio Forte^3^, Alessandro Priolo^1^, Massimiliano Lanza^1^, Marco Bella^1^, Luisa Biondi^1^, Antonio Natalello^1^

^1^Dipartimento di Agricoltura, Alimentazione e Ambiente (Di3A), University of Catania, via Santa Sofia 100, 95123, Catania, Italy

^2^Instituto de Ganadería de Montaña (CSIC-University of León), Finca Marzanas s/n, 24346 Grulleros, León, Spain

^3^Department of Veterinary Sciences, University of Turin, Largo Paolo Braccini 2, Grugliasco (TO), Italy

* Corresponding author: Gonzalo Hervás [g.hervas@csic.es](mailto:g.hervas@csic.es)

| **Supplementary Table S1.** Effect of dietary treatments on muscle fatty acid composition (mg/ 100 g of muscle). | | | | | | |
| --- | --- | --- | --- | --- | --- | --- |
|  | Diet*^a^* | | | |  |  |
|  | C | L | H | L+H | SED*^b^* | *P*-value |
| 10:0 | 2.45 ab | 2.88 ab | 2.03 b | 3.57 a | 0.507 | 0.024 |
| 12:0 | 2.80 ab | 2.91 ab | 2.05 b | 3.51 a | 0.510 | 0.048 |
| 14:0 | 43.4 ab | 44.3 ab | 30.4 b | 52.5 a | 7.18 | 0.028 |
| *c*9 14:1 | 2.03 ab | 2.27 ab | 1.41 b | 2.47 a | 0.390 | 0.048 |
| *t*9 14:1 | 0.121 xy | 0.119 xy | 0.062 y | 0.196 x | 0.047 | 0.052 |
| 15:0 | 7.40 | 7.16 | 4.18 | 7.21 | 1.443 | 0.085* |
| 15:0 *anteiso* | 1.41 | 1.56 | 0.898 | 1.77 | 0.320 | 0.053* |
| 15:0 *iso* | 0.863 | 0.786 | 0.522 | 0.957 | 0.204 | 0.172 |
| *t*10 15:1 | 1.66 | 1.45 | 1.24 | 1.88 | 0.248 | 0.072* |
| 16:0 | 379 ab | 374 ab | 271 b | 416 a | 50.4 | 0.035 |
| *c*9 16:1 | 29.2 a | 28.1 ab | 17.1 b | 28.3 ab | 4.21 | 0.018 |
| *t*9 16:1 | 0.232 b | 0.400 ab | 0.260 b | 0.550 a | 0.102 | 0.011 |
| 17:0 | 28.8 x | 24.0 xy | 14.5 y | 22.2 xy | 5.20 | 0.058 |
| 17:0 *anteiso* | 8.73 a | 7.49 ab | 4.78 b | 7.99 ab | 1.210 | 0.011 |
| 17:0 *iso* | 5.35 | 4.72 | 3.77 | 5.45 | 0.724 | 0.085* |
| *c*9 17:1 | 20.4 a | 15.8 ab | 8.07 b | 12.8 ab | 3.45 | 0.007 |
| 18:0 (SA)^c^ | 196 | 194 | 173 | 257 | 31.5 | 0.054* |
| *c*6 18:1 | 4.88 b | 9.63 ab | 5.97 b | 14.3 a | 1.885 | <0.001 |
| *c*9 18:1 (OA)^c^ | 643 | 598 | 496 | 700 | 89.8 | 0.141 |
| *c*11 18:1 | 30.8 | 28.9 | 23.5 | 27.6 | 3.33 | 0.163 |
| *t*6+7+8 18:1 | 2.26 b | 4.16 ab | 3.01 b | 6.63 a | 0.979 | <0.001 |
| *t*9 18:1 | 4.76 b | 6.02 ab | 5.23 b | 8.93 a | 1.069 | 0.001 |
| *t*10 18:1 | 47.4 ab | 80.7 a | 26.1 b | 41.9 b | 13.69 | 0.003 |
| *t*11 18:1 (VA)^c^ | 5.91 b | 10.1 ab | 7.34 b | 15.6 a | 2.14 | <0.001 |
| *c*9*c*12 18:2 (LA)^c^ | 112 ab | 98.6 b | 118 ab | 130 a | 8.92 | 0.011 |
| *c*9*t*11 18:2 (RA)^c^ | 5.37 b | 7.01 ab | 4.86 b | 8.82 a | 1.166 | 0.006 |
| *c*6*c*9*c*12 18:3 | 1.54 a | 1.09 b | 1.21 ab | 1.25 ab | 0.151 | 0.035 |
| *c*9*c*12*c*15 18:3 (αLNA)^c^ | 6.25 b | 30.7 a | 5.28 b | 28.2 a | 2.83 | <0.001 |
| *t*7 19:1 | 2.13 b | 8.94 a | 1.52 b | 8.73 a | 1.235 | <.0001 |
| 20:0 | 1.21 ab | 1.30 ab | 1.16 b | 1.87 a | 0.246 | 0.019 |
| *c*11 20:1 | 2.30 | 2.26 | 2.13 | 2.86 | 0.382 | 0.232 |
| *c*11*c*14 20:2 | 1.42 a | 0.804 b | 1.23 ab | 1.23 ab | 0.188 | 0.018 |
| 20:3 n-6 | 2.97 | 2.37 | 2.68 | 2.73 | 0.335 | 0.371 |
| 20:5 n-3 | 1.97 b | 5.92 a | 2.13 b | 5.51 a | 0.513 | <0.001 |
| 22:4 n-6 | 2.78 a | 1.45 b | 2.47 a | 1.65 b | 0.281 | <0.001 |
| 22:5 n-6 | 0.791 | 0.578 | 1.14 | 0.826 | 0.230 | 0.120 |
| 22:5 n-3 | 5.04 b | 8.32 a | 5.00 b | 8.46 a | 0.925 | <0.001 |
| 22:6 n-3 | 1.58 b | 2.37 ab | 1.66 b | 3.04 a | 0.360 | 0.001 |
| 23:0 | 2.26 | 1.93 | 2.20 | 2.49 | 0.268 | 0.233 |
| Totals | 1619 ab | 1623 ab | 1256 b | 1848 a | 209 | 0.049 |
| SFA^c^ | 679 ab | 667 ab | 511 b | 783 a | 93.9 | 0.043 |
| MUFA^c^ | 797 | 796 | 599 | 873 | 110.3 | 0.087* |
| PUFA^c^ | 142 b | 159 ab | 146 b | 192 a | 13.7 | 0.003 |
| OBCFA^c^ | 54.8 a | 47.7 ab | 30.8 b | 48.0 ab | 8.5 | 0.043 |
| ∑ n-3 | 14.8 b | 47.3 a | 14.1 b | 45.2 a | 4.29 | <0.001 |
| ∑ n-6 | 122 ab | 105 b | 127 ab | 138 a | 9.5 | 0.014 |

a,b,c Within a row, different superscripts indicate significant differences (P < 0.05).

x,y Within a row, different superscripts indicate a trend towards significant differences (0.05 ≤ P < 0.10), either for the general statistical model or after Bonferroni’s correction.

*^a^*C: Control diet; L: Linseed diet; H: Hazelnut skin diet; and L+H: Linseed + Hazelnut skin diet.

*^b^*SED: Standard error of the difference.

*^c^*SA: stearic acid; OA: oleic acid; VA: vaccenic acid; LA: linoleic acid; RA: rumenic acid; αLNA: α-linoleic acid; SFA: saturated fatty acid; MUFA: monounsaturated fatty acid; PUFA: polyunsaturated fatty acid; OBCFA: odd- and branched-chain fatty acid.

*In the pairwise analysis, no significant differences (P > 0.10) were found after adjustment for multiple comparisons using Bonferroni’s correction.
